# Genome-wide association study for birth weight in Nellore cattle points to previously described orthologous genes affecting human and bovine height

**DOI:** 10.1186/1471-2156-14-52

**Published:** 2013-06-13

**Authors:** Yuri T Utsunomiya, Adriana S do Carmo, Roberto Carvalheiro, Haroldo HR Neves, Márcia C Matos, Ludmilla B Zavarez, Ana M Pérez O’Brien, Johann Sölkner, John C McEwan, John B Cole, Curtis P Van Tassell, Flávio S Schenkel, Marcos VGB da Silva, Laercio R Porto Neto, Tad S Sonstegard, José F Garcia

**Affiliations:** 1 Faculdade de Ciências Agrárias e Veterinárias, UNESP - Univ Estadual Paulista, Jaboticabal, São Paulo, 14884-900, Brazil; 2 GenSys Consultores Associados, Porto Alegre, 90680-000, Brazil; 3 Department of Sustainable Agricultural Systems, Division of Livestock Sciences, BOKU - University of Natural Resources and Life Sciences, Vienna, Austria; 4 Centre for Reproduction and Genomics, AgResearch, Invermay, Mosgiel, New Zealand; 5 ARS-USDA - Agricultural Research Service - United States Department of Agriculture, Animal Improvement Programs Laboratory, Beltsville, Maryland, 20705, USA; 6 United States Department of Agriculture, Agricultural Research Service, Bovine Functional Genomics Laboratory, Beltsville, Maryland, 20705, USA; 7 Centre for Genetic Improvement of Livestock, University of Guelph, Guelph, ON N1G2W1, Canada; 8 Bioinformatics and Animal Genomics Laboratory, Embrapa Dairy Cattle, Juiz de Fora, Minas Gerais, Brazil; 9 School of Veterinary Sciences, The University of Queensland, Gatton, QLD 4343, Australia; 10 School of Rural and Environmental Science, University of New England, Armidale, NSW 2351, Australia; 11 Faculdade de Medicina Veterinária de Araçatuba, UNESP – Univ Estadual Paulista, Araçatuba, São Paulo, 16050-680, Brazil

**Keywords:** GWAS, Birth weight, *Bos primigenius indicus*, Nellore cattle, Stature

## Abstract

**Background:**

Birth weight (BW) is an economically important trait in beef cattle, and is associated with growth- and stature-related traits and calving difficulty. One region of the cattle genome, located on *Bos primigenius taurus* chromosome 14 (BTA14), has been previously shown to be associated with stature by multiple independent studies, and contains orthologous genes affecting human height. A genome-wide association study (GWAS) for BW in Brazilian Nellore cattle (*Bos primigenius indicus*) was performed using estimated breeding values (EBVs) of 654 progeny-tested bulls genotyped for over 777,000 single nucleotide polymorphisms (SNPs).

**Results:**

The most significant SNP (rs133012258, P_GC_ = 1.34 × 10^-9^), located at BTA14:25376827, explained 4.62% of the variance in BW EBVs. The surrounding 1 Mb region presented high identity with human, pig and mouse autosomes 8, 4 and 4, respectively, and contains the orthologous height genes *PLAG1*, *CHCHD7*, *MOS*, *RPS20*, *LYN*, *RDHE2* (*SDR16C5*) and *PENK*. The region also overlapped 28 quantitative trait loci (QTLs) previously reported in literature by linkage mapping studies in cattle, including QTLs for birth weight, mature height, carcass weight, stature, pre-weaning average daily gain, calving ease, and gestation length.

**Conclusions:**

This study presents the first GWAS applying a high-density SNP panel to identify putative chromosome regions affecting birth weight in Nellore cattle. These results suggest that the QTLs on BTA14 associated with body size in taurine cattle (*Bos primigenius taurus*) also affect birth weight and size in zebu cattle (*Bos primigenius indicus*).

## Background

Birth weight (BW) is an economically important trait in beef cattle, and is usually the first characteristic measured in a calf. Birth weight is associated with growth-related traits [1], mature size [2] and carcass weight, thus being a valuable production indicator, as well as a selection criterion to improve calving ease [3,4].

Despite the beef industry’s pursuit of animals with rapid growth, yielding heavier carcasses, selection for these objectives needs to be properly balanced against selection for reproductive traits, which have great economic importance in beef cattle production systems [5,6]. While low estimated breeding values (EBVs) for BW are associated with reduced calf viability [4] and lower growth rates [3,7], the use of sires with high EBVs for BW on dams with small pelvic size may result in higher rates of dystocia [7] and increased perinatal mortality [8]. These antagonisms result from the strong association of birth weight with the body size of the calf, i.e., with the stature of the animal [2]. Calving difficulties can result from a mismatch between pelvic opening and calf size [6]. This relationship between BW with reproductive and growth/size traits highlights the importance of understanding the underlying genetic architecture of BW.

Birth weight exhibits sufficient variability and heritability in the Nellore breed (*Bos primigenius indicus*), with an average of 29.8 ± 2.7 kg [9] and estimated heritability between 0.25 and 0.33 [1,9,10]. Despite the low frequency of dystocia in Nellore cows, BW has been recorded and used to monitor genetic trend. One selection strategy of the breeding programs in Brazil has been to preferentially use sires with higher EBVs for weaning and yearling weights, but with low or close to average EBVs for BW [11]. The identification of major genes and variants affecting multiple weight and carcass traits or influencing BW alone would be of help to balance these conflicting goals, because BW is positively correlated with weaning and yearling weights [1].

In the past two decades, linkage studies attempting to map quantitative trait loci (QTLs) affecting weight, growth, or stature in cattle have been published (e.g. [12-16]). The release of the reference bovine genome [17], the discovery of common single nucleotide polymorphisms (SNPs) across breeds [18,19], and the availability of high-throughput microarrays have enhanced the process of mapping loci that affect complex traits. This has led to several population-based investigations of associations between weight/growth/height phenotypes with genome-wide variants in different cattle breeds [15,20-23].

In particular, two regions of the bovine genome associated with stature and growth have been highlighted recently. The first, located on *Bos primigenius taurus* (BTA) autosome 6 [20,23], shelters the orthologous genes *NCAPG* and *LCORL*, which have been also found to be associated with adult height in humans [24,25]. The second, located on BTA14 [15,21-23], contains the genes *PLAG1*, *CHCHD7*, *RDHE2*, *MOS*, *RPS20*, *LYN*, *PENK* and *TGS1*, that were previously found to affect stature in both cattle and humans [22,24,26-28]. Importantly, the majority of the genome-wide association studies (GWAS) reported in literature were conducted in the humpless subspecies of cattle (*Bos primigenius taurus*, known as taurine cattle), and GWAS in the humped bovine subspecies (*Bos primigenius indicus*, often referred as indicine or zebu cattle) are only now emerging, especially because the first SNP microarrays were optimized for taurine cattle [19].

In this paper, results from a genome-wide scan for SNPs associated with BW variation in Nellore cattle using EBVs of progeny-tested Brazilian bulls are reported. As EBVs take into account information from performance of the individual, progeny and parents, pedigree relationships, and systematic management and environmental factors, they can be used as composite phenotypes for proceeding with association analyses (see, e.g., [29]). The objective of this study was to identify putative SNP associated with differences in BW and to explore the genomic regions around them to unravel prospective functional relationships among weight, fertility, and growth/size traits.

## Results

### Genotype informativeness and quality control

From the initial set of 777,961 SNPs, 42,669 (5.5%) were non-autosomal markers. Fifty four autosomal SNPs with redundant genomic coordinates were identified and excluded from further analyses. The identity by state (IBS) check revealed no unexpected sample duplicates. A total of 223,309 (30.4%) markers were excluded due to minor allele frequency (MAF) < 0.02. The number of SNPs excluded due to SNP Call Rate (CR_SNP_) < 0.98 and Fisher’s exact test *P*-value for Hardy-Weinberg Equilibrium (HWE) < 1 × 10^-5^ were 122,611 (16.7%) and 13,194 (1.8%), respectively. Five individuals were removed due to low Call Rate (CR_IND_). The final dataset included data for 649 individuals and 434,020 SNPs.

### Descriptive statistics of dependent variables

Based on the results for the Shapiro-Wilk test, there was no evidence that EBVs deviated from normality (*P* = 0.415), and no outliers were observed. Average accuracy was 0.87 ± 0.11, with minimum, median and maximum accuracies of 0.51, 0.91 and 0.99, respectively. After fitting the regression in formula (**2**), there was no evidence against the hypotheses of normally distributed (*P* = 0.57) and homoskedastic residuals (studentized Breusch-Pagan test, *P* = 0.11). These findings suggest that the dependent variables used were reliable and did not violate possible assumptions of the statistical analyses used hereafter.

### Population substructure

**Figure 1:**
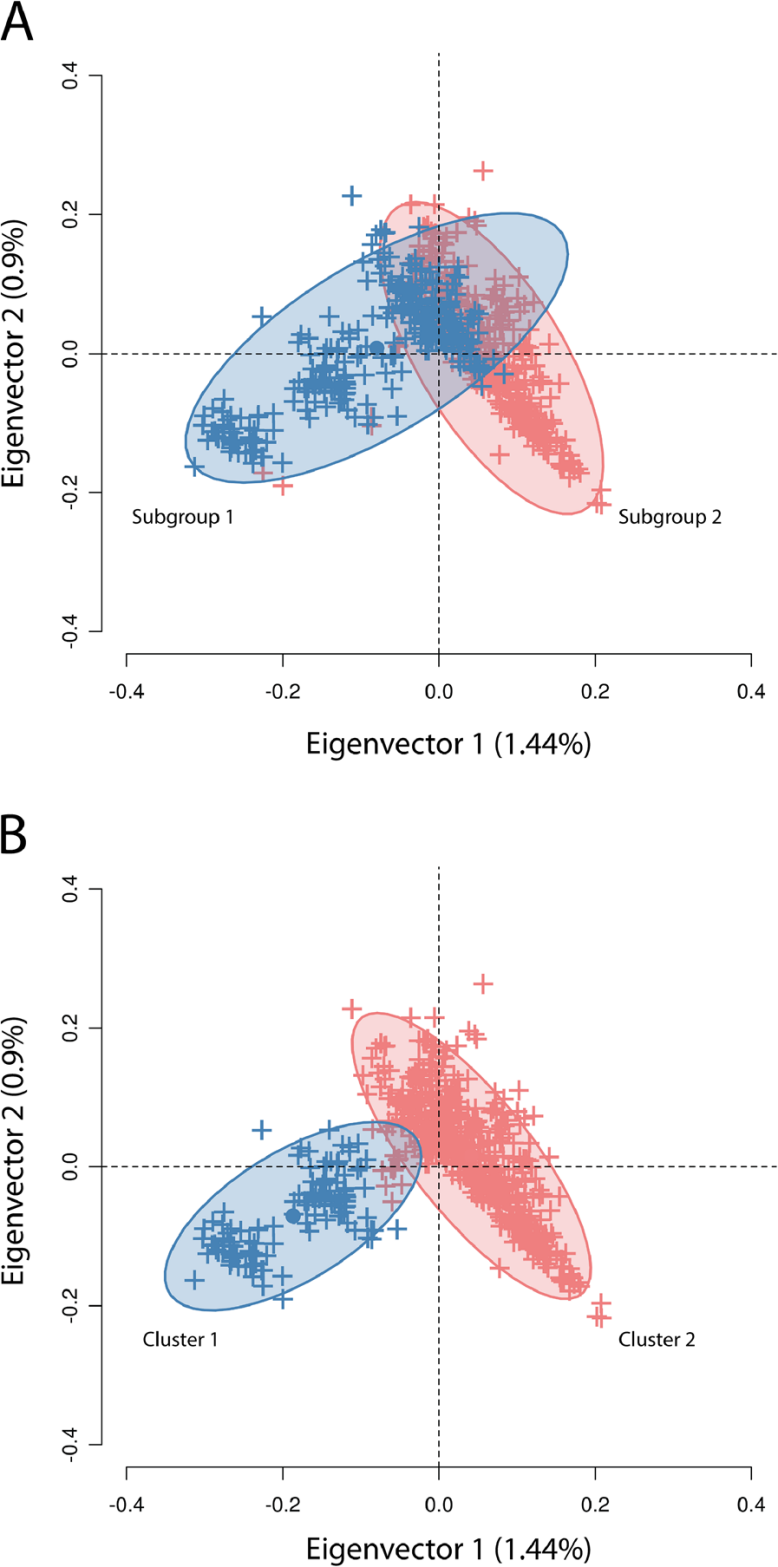
**Principal Coordinates Analysis based on the genomic kinship coefficient.** Percentages inside brackets correspond to the variance explained by each respective eigenvector. Each ‘+’ represents an individual and ovals are 95% inertia ellipses. **A**) Subjects colored according to breeding program subgroups. **B**) Subjects colored according to k-means clustering results.

The Principal Coordinates Analysis (PCoA) revealed genetic stratification among the Nellore samples (Figure 1). After using k-means clustering to assign individuals to two different groups according to their coordinates in the PCoA, a highly significant association (*P* = 5.41 × 10^-34^) between the k-means assignments (n_cluster1_ = 531, n_cluster2_ = 118) and breeding program subgroups (n_subgroup1_ = 352, n_subgroup2_ = 297) was found. For the k-means groups, BW average was 0.434 ± 1.306 in cluster 1 and -0.216 ± 1.295 in cluster 2. For the breeding program subgroups, the trait averages in subgroup 1 and subgroup 2 were 0.683 ± 1.276 and -0.119 ± 1.255, respectively. When considering either k-means clusters or breeding program subgroups as subpopulation labels, the EBVs showed homogeneity of variance (*P* > 0.05), but the trait mean was significantly different between groups (*P* = 1.21 × 10^-6^ and *P* = 4.37 × 10^-15^ for k-means and breeding program, respectively). Thus, population substructure was a potential confounder in the genotype-EBV association analysis, which justified the inclusion of eigenvectors from the PCoA as fixed effects in the linear model.

### Association analysis

**Figure 2:**
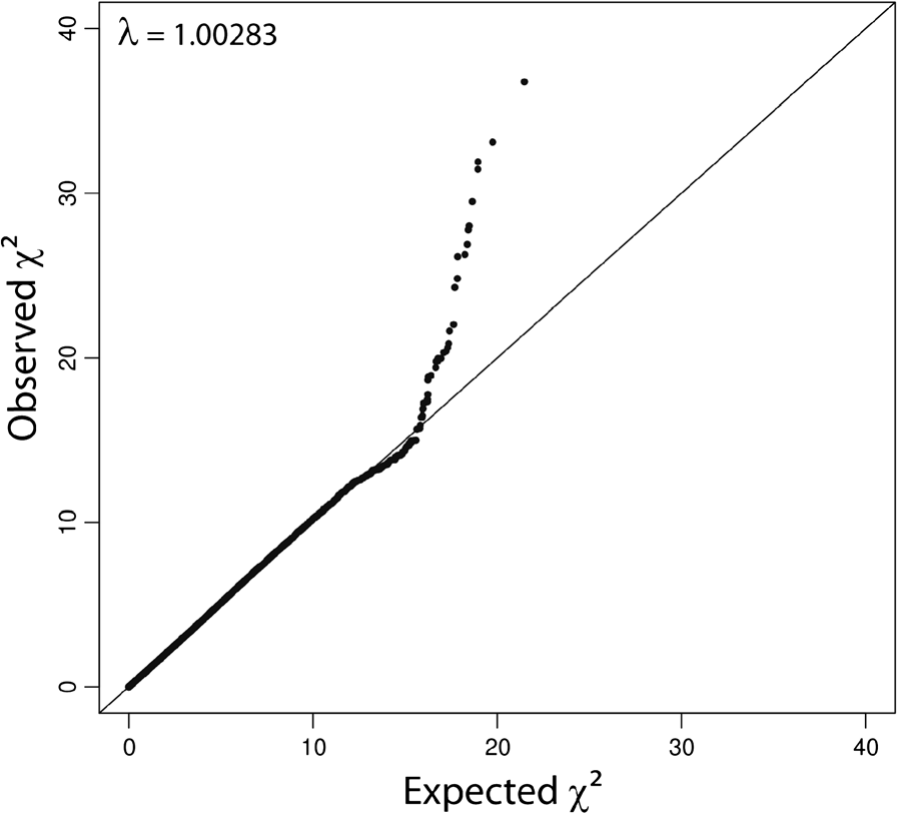
**Quantile**-**quantile plot for the test statistics (χ**^**2**^**) used in the association analysis.**

**Figure 3:**
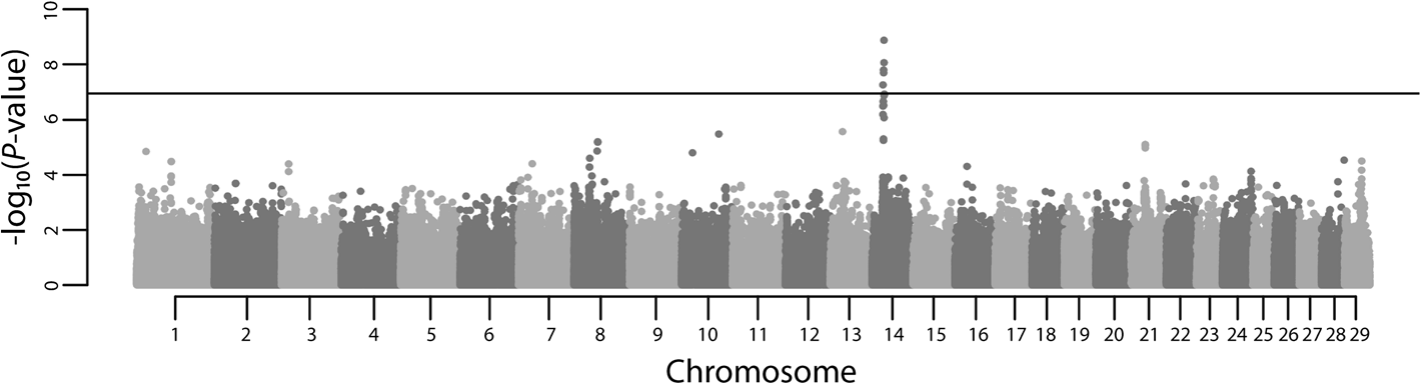
**Manhattan plot of genome-wide -log**_**10**_**(*P***-**values) for birth weight estimated breeding values in Nellore cattle.** The horizontal line represents the Bonferroni significance threshold (α = 1.15 × 10^-7^).

A total of 32 eigenvectors from the PCoA were significantly correlated with the phenotypes, which together explained 15.23% of the genotypic variability. Residuals from the weighted regression on these significant eigenvectors were used as the dependent variable for the SNP association analysis. The quantile-quantile (Q-Q) plot (Figure 2) showed that the deviation of the observed test statistics from the theoretical quantiles was mild and acceptable (λ = 1.002831), and the values were adjusted for the inflation factor via Genomic Control (GC). The *T*^2^ values deviating from the expected values were interpreted as SNPs departing from the null hypothesis. The genome-wide deflated *P*-values are shown in Figure 3.

**Figure 4:**
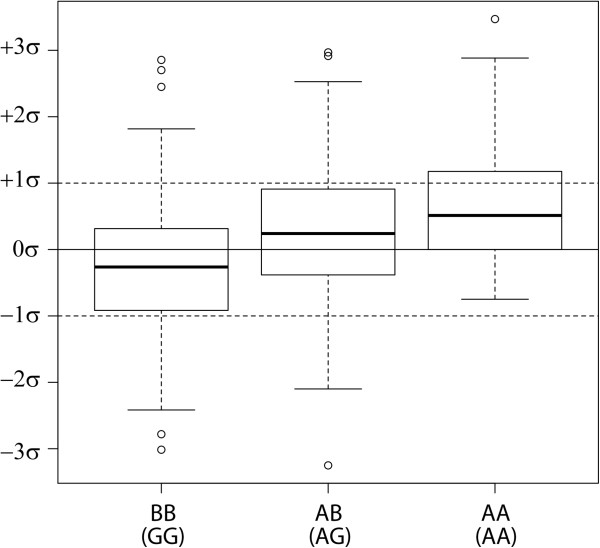
**Box plots for the birth weight estimated breeding values according to rs133012258 genotypes.** Values in the y axis are expressed in terms of standard units.

A peak crossing the boundary for Bonferroni significance (α = 1.15 × 10^-7^) was detected on BTA14, comprising 5 SNPs (Table 1) which were highly linked (mean r^2^ = 0.728 ± 0.12). The most significant SNP (rs133012258, *P*_GC_ = 1.34 × 10^-9^), located at BTA14:25376827, had an estimated allele substitution effect of 0.452 kg (i.e., for each extra A allele, the BW breeding value is expected to increase 0.452 kg), with lower and upper limits for the 95% confidence interval (CI) of 0.306 kg and 0.598 kg, respectively, and the percentage of the variance in sires EBVs explained by the SNP was 4.62% (with a 95% CI of 2.12-8.09%). The overall rs133012258 A allele frequency was 0.274, whereas the breeding program subgroups 1 and 2 had frequencies of 0.351 and 0.184, respectively. Figure 4 shows the distribution of the EBVs (in standard deviations) for the three genotype classes of rs133012258. In both Illumina TOP and Forward allele notation, the AB correspondence for rs133012258 was A = A and B = G.

**Table 1:** Summary of parameters and statistics estimated for the identified significant SNPs

**Ensembl variant ID**	**Illumina probe ID**	**BTA14 Position (bp)**	**n**	**Effect allele**	**Allele frequency**	**CR **^**a **^**(%)**	**HWE *P*-value**	**β**^**b **^**(kg)**	**SE**	***T***^**2**^^**c**^	**GC-*T***^**2**^^**d**^	***P*-value**
rs133012258	BovineHD1400007343	25376827	649	A	0.274	100.00	0.920	0.452	0.074	36.858	36.753	1.34×10^-9^
rs41627948	BovineHD1400007374	25504073	648	B	0.181	99.85	0.110	0.522	0.090	33.202	33.108	8.72×10^-9^
rs42646720	BovineHD1400007144	24590812	649	B	0.243	100.00	0.390	0.457	0.081	31.999	31.909	1.62×10^-8^
rs136764901	BovineHD1400007159	24651537	641	B	0.244	98.77	0.200	0.459	0.082	31.546	31.457	2.04×10^-8^
rs136287861	BovineHD1400006765	23313228	645	A	0.272	99.38	0.766	0.408	0.075	29.580	29.497	5.60×10^-8^

^a^*CR* Call rate.

^b^*β* Estimated allele substitution effect.

^c^*T*^2^ Chi-squared statistics for β: $\chi^{2}=T^{2}=\frac{\beta^{2}}{SE^{2}}$.

^d^*GC-T*^*2*^ Corrected Chi-squared statistics for β. Genomic control correction was performed by dividing the χ^2^ statistics by the distribution inflation/deflation factor estimate (λ = 1.002831).

**Figure 5:**
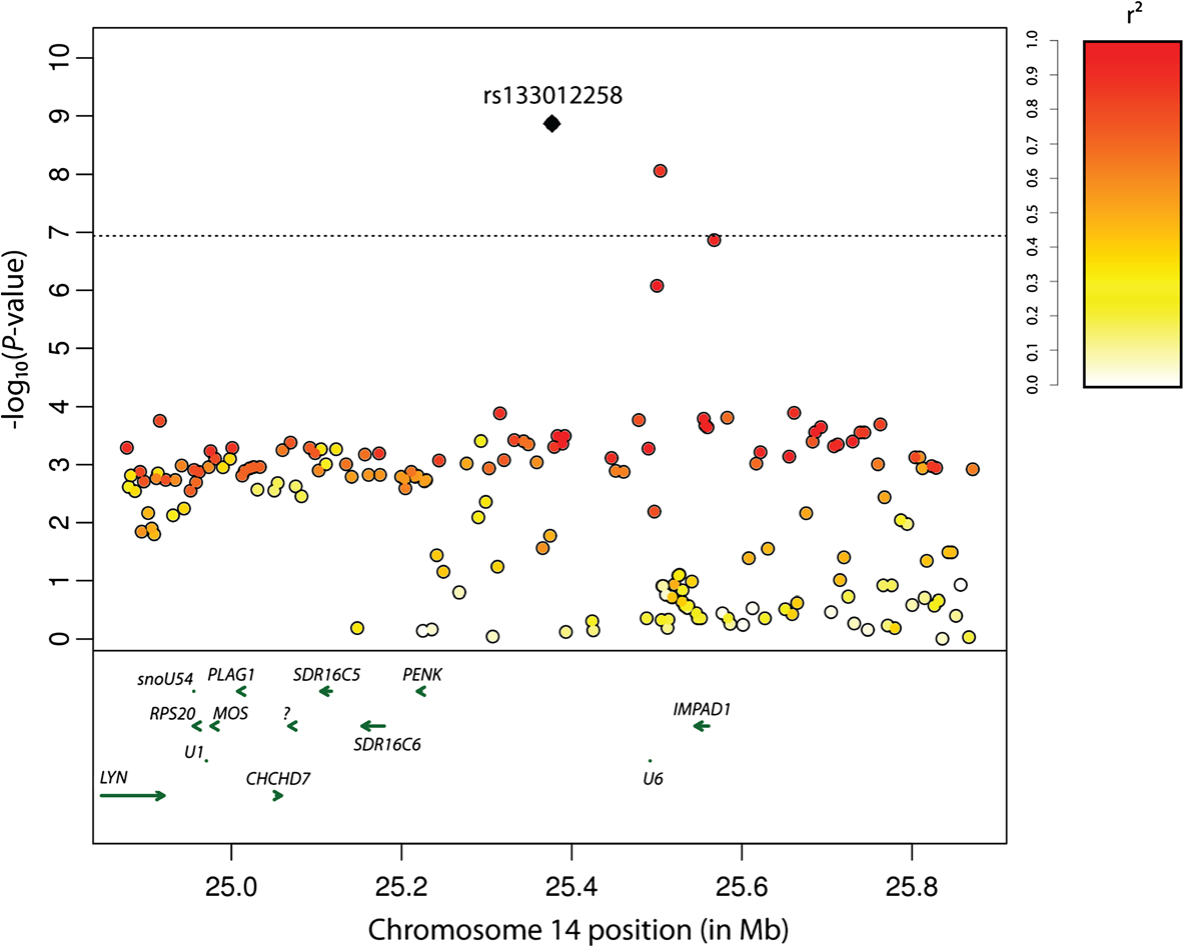
**Regional association plot for birth weight in the 1 Mb window around rs133012258.** Upper box: each dot represents a SNP, and its color heat the degree of linkage disequilibrium with rs133012258 (black diamond). The horizontal dashed line represents the Bonferroni significance threshold (α = 1.15 × 10^-7^). Lower box: genes (green arrows; right-handed = positive strand, left-handed = negative strand) within the region in the UMD v3.1 assembly.

**Figure 6:**
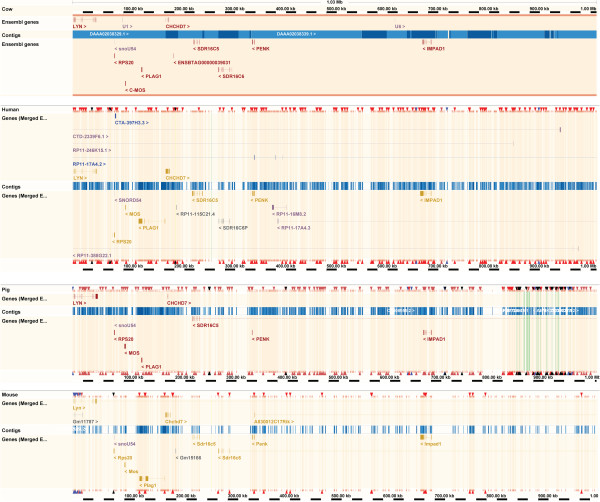
**Ensembl alignments of UMD v3.1 sequence for the 1 Mb region surrounding rs133012258.** The bovine reference genome sequence was aligned against (from top to bottom) the human (GRCh37 assembly), pig (Sscrofa10.2 assembly) and mouse (GRCm38 assembly) genome builds. Gene colors: yellow - merged Ensembl/Havana, red - protein coding, blue - processed transcript, grey - pseudogene, purple - RNA gene. Triangles: black - breakpoint between different chromosomes, blue - inversion in chromosome, brown - breakpoint on chromosome, red - gap between two underlying slices.

One of the 5 significant SNPs, rs42646720 (BTA14:24590812, *P*_GC_ = 1.62 × 10^-8^), is located within intron 2 of the gene Kell blood group complex subunit-related family, member 4 (*XKR4* or *KIAA1889*, ENSBTAG00000044050). Thirteen genes were found within 500 kb of the most significant SNP (Figure 5), including the human height-associated orthologous genes *PLAG1*, *CHCHD7*, *MOS*, *RPS20*, *LYN*, *RDHE2* (*SDR16C5*) and *PENK* (Table 2). The bovine reference genome sequence of this region was found to have high identity with human (*Homo sapiens* - HSA), pig (*Sus scrofa* - SSC) and mouse (*Mus musculus* - MMU) autosomes 8 (HSA8), 4 (SSC4) and 4 (MMU4), respectively (Figure 6), and the majority of the genes had homology across species (Table 2). The most significant SNP also overlapped 28 QTLs previously reported in the literature by linkage mapping studies using different cattle breeds (Table 3), including QTLs for birth weight, mature height, carcass weight, stature, pre-weaning average daily gain, calving ease and gestation length.

**Table 2:** **List of genes within the 1 Mb region surrounding the most significant SNP** (**rs133012258**)

**Gene**	**Ensembl ID**	**BTA14 coordinates**	**Distance from SNP (kb)**	**Strand**	**HSA8 homology**	**SSA4 homology**	**MMA4 homology**	**Description**
U6	ENSBTAG00000043923	25492090:25492184	115.4	+	No homologues	No homologues	No homologues	U6 spliceosomal RNA
PENK	ENSBTAG00000004924	25218586:25222991	153.8	-	ENSG00000181195	ENSSSCG00000006243	ENSMUSG00000045573	Proenkephalin-A
IMPAD1	ENSBTAG00000015637	25544907:25560879	168.1	-	ENSG00000104331	ENSSSCG00000006242	ENSMUSG00000066324	Inositolmonophosphatase 3
SDR16C6	ENSBTAG00000040321	25153583:25179651	197.2	-	No homologues	No homologues	ENSMUSG00000071019	Short-chain dehydrogenase/reductase family 16C member 6
SDR16C5 (RDHE2)	ENSBTAG00000018570	25105062:25117554	259.3	-	ENSG00000170786	ENSSSCG00000006245	ENSMUSG00000028236	Epidermal retinol dehydrogenase 2
Unknown	ENSBTAG00000039031	25067486:25067823	309.0	-	No homologues	No homologues	No homologues	Uncharacterizedprotein
CHCHD7	ENSBTAG00000033284	25052885:25058779	318.0	+	ENSG00000170791	ENSSSCG00000006246	ENSMUSG00000042198	Coiled-coil-helix-coiled-coil-helix domain-containing protein 7
PLAG1	ENSBTAG00000004022	25007291:25009296	367.5	-	ENSG00000181690	ENSSSCG00000006247	ENSMUSG00000003282	Pleiomorphic adenoma gene 1
MOS	ENSBTAG00000019145	24975950:24976948	399.9	-	ENSG00000172680	ENSSSCG00000006248	ENSMUSG00000078365	V-mosMoloneymurine sarcoma viral oncogenehomolog
U1	ENSBTAG00000028889	24970516:24970679	406.1	+	No homologues	No homologues	No homologues	U1 spliceosomal RNA
RPS20	ENSBTAG00000019147	24955079:24956324	420.5	-	ENSG00000008988	ENSSSCG00000006249	ENSMUSG00000028234	40S ribosomalprotein S20
snoU54	ENSBTAG00000045097	24955769:24955835	421.0	-	No homologues	No homologues	No homologues	Small nucleolar RNA U54
LYN	ENSBTAG00000020034	24847257:24920713	456.1	+	ENSG00000254087	ENSSSCG00000006250	ENSMUSG00000042228	Tyrosine-proteinkinaseLyn

**Table 3:** **QTLdb hits within the 1 Mb region surrounding the most significant SNP** (**rs133012258**)

**Trait**	**BTA14 coordinates**	**QTLdb ID**	**PubMed ID**
Body weight (birth)	6311565:71762521	5375	19016677
Height (mature)	19204282:42398519	10962	20477797
Carcass weight	25224396:30870876	1375	16151698
	10808022:28658498	10960	20477797
Stature	25219037:65017465	4613	10575619
Pre-weaning average daily gain	25224396:35530275	2630	15537758
Calving ease (maternal)	19204282:28658498	10959	20477797
Gestation length	6311565:30372479	5374	19016677
	17512260:48646289	5385	19016677
Rump angle	25224396:29928419	1592	16230715
Longissimus muscle area	19204282:42398519	10964	20477797
Fat thickness at the 12th rib	19204282:28658498	10961	20477797
Marbling score	16670076:73064076	1334	14677852
Abnormal flavor intensity	5565085:28658498	4833	18254735
Tick resistance	5545944:77366190	9917	17894560
Milk yield	25372161:25525285	6209	18650300
	16243029:28716621	3608	12729552
Milk fat yield (or percentage)	13401044:35992517	2733	9691050
	1641277:25448723	3408	12605852
	25224396:29928419	2676	14762090
	13401044:35992517	2732	9691050
Milk protein yield (or percentage)	1641277:56300551	3413	12605852
	9479897:44651695	2604	12778594
	1641277:81189386	10099/10100/10101	18298934
Somatic cell score	13401044:35992517	2734	9691050
	13401044:35992517	2776	14556700
	20567087:44651695	4884	17954769
Clinical mastitis	9479131:44651695	3177	14762087

## Discussion

Five SNPs on BTA14 were identified as associated with BW in Nellore cattle (*P* < 1.15 × 10^-7^), whose surrounding region has been shown to contain many QTLs, genes and variants affecting stature-related traits in cattle by several independent studies [12-15,21-23]. More particularly, the genes *PLAG1*, *CHCHD7*, *RDHE2*, *MOS*, *RPS20*, *LYN* and *PENK* have been found to influence both human and cattle height [22-28].

The BTA14 region pointed out by the present study has also been shown to be associated with reproductive traits. Cole *et al*. [16] reported a QTL on BTA14 associated with stillbirth, which also has been associated with body size in dairy cattle [8,30], but found no effect on stature or other conformation traits on that chromosome. The region also associates with many fertility and growth-related traits in the indicine breed Brahman, for example scrotal circumference [31,32], age at the first *corpus luteum*[31,33], blood levels of insulin-like growth factor 1 (*IGF1*) [32,33] and hip height [33].

A significant SNP was found within intron 2 of the *XKR4* gene in the present study. Lindholm-Perry *et al*. [34] identified five SNPs near *XKR4* associated with feed intake and gain in crossbred steers. Bolormaa *et al*. [35] found five SNPs in a narrow region of BTA14 encompassing *XKR4* associated with rump fat thickness measured at the P8 position (CHILLP8) in seven breeds of cattle, including taurine, indicine and composite breeds. The authors found that four of these SNPs were also associated with CHILLP8 in a confirmatory sample of 1,338 animals, including Angus, Hereford and Brahman cattle. Furthermore, Porto Neto *et al*. [36] performed a replication study using samples of Belmont Red, Santa Gertrudis and Brahman animals genotyped for SNPs within *XKR4* and found that although the SNP effect may vary depending on the breed, the variant rs42646708 (BTA14:24573257) explain around 1.3% of CHILLP8 variance in cattle. This SNP is also located within intron 2 of *XKR4*, only 17.6 kb apart from the intronic SNP detected in the present study, which strongly suggests *XKR4* as a candidate gene for being further explored in future studies of weight and carcass traits in Nellore cattle.

The most significant SNP (rs133012258, *P*_GC_ = 1.34 × 10^-9^) was found to explain 4.62% of the variance in sires EBVs, with a 95% CI of 2.12-8.09%. One hundred and eighty loci associated with human adult height explain only 10% of the phenotypic variance together, while individual loci account for 0.4% or less [37]. SNPs analyzed by [22] within a nearby BTA14 region explain from 0.29 to 2.53% of the bovine stature variability, and the quantitative trait nucleotides (QTN) spanning *MOS*, *CHCHD7* and *PLAG1* described by [27] explain from 1.10 to 3.50% of height in Jersey and Holstein breeds. Furthermore, the genome-wide survey performed by [21] provided strong evidence for two QTL on BTA14 and BTA21 that together explain at least 10% of the variation of EBVs for calving ease in the German Fleckvieh.

Considering that multiple stature-related traits are governed by variants with small effects, and that the genomic region identified in this study has been previously found to be associated with several of these traits, the putative SNP detected in the present analysis can be considered as a marker in linkage disequilibrium (LD) with major untyped (i.e., not probed by the SNP assay used) causative variants affecting BW and other height-associated traits in Nellore cattle, and further studies would be needed to determine if the QTNs reported by [27] are also segregating in the Nellore population. Also, future investigations are needed to better characterize the effect of nearby SNPs on other weight and carcass traits in Nellore cattle, as it is not clear yet how the putative pleiotropic effect of these variants would be used towards balancing conflicting selection goals for birth, weaning and yearling weights. Although we cannot confirm that the allele substitution effects of these SNPs work in the same direction for all three traits, because only birth weight was analyzed here, these findings suggest that the SNPs identified would be key polymorphisms to be monitored over time. In a scenario where the SNP effects have the same direction in all three traits, one strategy could be avoiding strong positive selection or drifting of the allele that contributes to higher BW EBVs, and identify and promote positive selection of other variants that have effects on weaning and yearling weights only.

The high identity found in the alignment of this BTA14 region against other mammalian species genomes suggests that these orthologous genes are located in a conserved syntenic block which may have arisen and been maintained after speciation from a common ancestor of the mammal clade. Moreover, the evidence for variants associated with growth and stature within this BTA14 region in both taurine and zebu cattle raises two hypotheses: 1) these variants have been introgressed into Nellore via historical admixture with taurine Creole cattle in the maternal line, and was maintained in the breed in spite of several generations of backcrossing; 2) these are ancient polymorphisms, probably already segregating in the founder population of wild Aurochs (*Bos primigenius*) before subspecies formation.

Regarding functional meaning, the set of genes reported participate in diverse growth and tumor development mechanisms. Among these genes, *PLAG1* is the most appealing functional candidate. It is an oncogene that encodes a transcription factor broadly expressed during fetal development, but is down-regulated at birth [27]. It interacts with several growth factors controlling body size, including *IGF2*[38]. In addition, *PLAG1* knock-out mice have been shown to have marked growth retardation and reduced fertility [39]. In a replication study, [28] confirmed the findings reported by [27], demonstrating association of growth rate and early life and peripubertal body weight with *PLAG1* polymorphisms, supporting its status as a key regulator of mammalian growth.

The lack of significant association between BW and SNPs within other previously described weight- and height-related chromosome regions in the present study should not be interpreted as a lack of existence of true association, but rather it might be due to limitations specific to this study. Firstly, because complex trait mapping requires large sample sizes and only 649 bulls were analyzed here. Secondly, the significance level adopted was highly stringent, which may have caused inflation of type II errors. In spite of these limitations, it was possible to demonstrate that a well-characterized chromosome region affecting human and taurine cattle stature also associates with BW in a zebu breed. The release of a *Bos primigenius indicus* reference genome assembly, as well as the application of re-sequencing and replication studies would help improve resolution to narrow down the genomic region as close as possible to the true causative variants.

## Conclusions

This study is believed to be the first genome-wide association study applying a high-density SNP panel to identify putative chromosome regions affecting birth weight in zebu cattle. The findings presented, which are strongly supported by the literature, point to orthologous genes already known to affect growth- and stature-related traits in both humans and cattle, which may shelter ancient polymorphisms responsible for variation in those traits since before cattle subspecies divergence.

## Methods

### Estimated breeding values

Estimated breeding values for BW were obtained from routine genetic evaluations using performance and pedigree data from the *Aliança* database [11], containing data from different commercial Nellore breeding programs, including more than 250 farms distributed across Brazil and Paraguay. The genetic evaluation for BW was calculated using a subset of that data that included 542,918 animals, born from 1985 to 2011, and distributed in approximately 5,000 distinct contemporary groups. These data were collected in 243 grazing-based herds in Brazil. Estimated breeding values were obtained using an animal model that included fixed effects for the age of dam at calving and contemporary group (defined as animals from the same herd, born in the same year and season, and belonging to the same management group at birth, and sex), as well as random effects that include direct additive genetic, maternal additive genetic, maternal permanent environmental and residual error effects. The variance ratios required to solve the mixed model equations were computed based on restricted maximum likelihood (REML) estimates of the variance components from previous studies in this population. Only EBVs of progeny-tested bulls whose accuracy (i.e., square root of reliability, calculated based on prediction error variance estimates) was ≥ 0.50 were used for sample collection and genotyping (described later). The majority of the bulls were used under artificial insemination service.

### Genotyping, informativeness, and quality assurance

A total of 654 progeny-tested Nellore bulls were genotyped with the Illumina® BovineHD Genotyping BeadChip assay, according to the manufacturer’s protocol. Genotype calls (i.e. successfully determined genotypes) were defined as genotypes with GenCall Scores greater than 0.70, using the validated standard cluster file provided by the manufacturer. As chromosomes X, Y and mtDNA present different mode of inheritance from the rest of the genome, only autosomal markers with unique genomic coordinates were included into the analyses. After this initial screening, potential duplicated samples were determined by calculating the proportion of alleles identical by state (IBS) shared between all pairs of individuals. Any pair of samples with IBS ≥ 0.95 for 2,000 randomly sampled markers was considered unexpected duplicates, and resulted in the exclusion of both members of the pair. Individual SNPs were removed from the dataset if they did not exhibit: 1) minor allele frequency (MAF) greater than or equal to 0.02, 2) Fisher’s exact test *P*-value for Hardy-Weinberg Equilibrium (HWE) greater than or equal to 1 × 10^-5^ (i.e. extremely deviating from HWE, suggesting potential genotyping error) or 3) Call rate (CR_SNP_) of at least 98%. After the SNP pruning, individuals exhibiting call rate (CR_IND_) below 90% were also removed. These procedures and many others described later were performed in the *R* v2.15.0 environment [40], using combinations of functions from the *R* base, locally developed scripts, and the GenABEL v1.7-2 package [41].

### Assessment of population substructure

Sires genotyped in this study were known to belong to one of two major breeding program subgroups in the *Aliança* database [11] that have different selection objectives. One group emphasizes selection for weaning and yearling weight (subgroup 1) and the other emphasizes selection for fertility and carcass traits (subgroup 2). Thus, genetic stratification was expected and therefore population substructure was evaluated by performing a Principal Coordinates Analysis (PCoA). Pair-wise genomic kinship coefficients for all subjects under study were calculated first, following [42,43]:

(1) $${\hat{f}}_{i,j}=\frac{1}{L}\sum_{l=1}^{L}\frac{\left(g_{l,i}-p_{l}\right)\left(g_{l,j}-p_{l}\right)}{p_{l}\left(1-p_{l}\right)}$$

Where ${\hat{f}}_{i,j}$ is the estimated genomic kinship between individuals i and j, *L* is the total number of loci used for the calculation, *p*_*l*_ is the reference allele frequency for locus *l*, and *g*_*l*,*i*_ and *g*_*l*,*j*_ are the locus *l* genotypes for individuals i and j, respectively (coded as 0, 1 or 2 reference alleles). Calculations were based on 10,000 randomly sampled markers using GenABEL [41]. The calculated genomic kinship coefficients within the yielded *n* x *n* symmetric matrix (where *n* is the total number of samples) were then transformed to squared Euclidean distances, and the dissimilarities between the subjects within the matrix were captured in *n*-1 dimensional spaces of *n* observations (eigenvectors), via classical multidimensional scaling [44].

A clustering analysis was applied to the two eigenvectors that explained the largest proportion of the data variance using the k-means algorithm [45] implemented in *R*[40]. Individuals were clustered into 2 groups, and the association between the prior information on breeding program and the k-means clustering results was tested using Pearson’s χ^2^ with Yates’ continuity correction in order to see if the algorithm could reproduce the known breeding programs subgroups. Additionally, an F-test for homogeneity of variance between subpopulations and a t-test for difference between subpopulation means, defining subpopulations either as k-means assignments or breeding program of origin, was performed to determine if there was confounding due to stratification.

### Association analysis

In order to reduce computation time, the ideas of [46] were abstracted and a three-step association analysis was performed. In the first step, a linear regression using the weighted least squares method with weights equal to the squared accuracy (i.e., reliability) of the EBVs was applied. By weighting the EBVs by their respective accuracies, the uncertainty around the estimates was taken into account when estimating the regression parameters. The following model was fitted:

(2) $$y_{i}=\mu +\sum_{j=1}^{n}\beta_{j}X_{\mathrm{ij}}+\epsilon_{i}$$

Where y_i_ is the EBV of sire i, μ is the overall mean, *X*_*ij*_ is value i (corresponding to sire i) in the eigenvector j calculated in the PCoA, *β*_*j*_ is the estimated effect of eigenvector j, and *ϵ*_*i*_ is the residual effect for animal j. Only eigenvectors significantly (P < 0.05) correlated with the dependent variable, as assessed by Pearson correlations, were included in the model. Next, the residuals were obtained from the fitted model in (**2**):

(3) $$y_{i}{}^{*}=y_{i}-{\hat{y}}_{i}$$

and had their homoskedasticity and normality tested by using the studentized Breusch-Pagan test and the Shapiro-Wilk test, respectively. Then, these residuals were used as the new dependent variable for a single-marker linear regression:

(4) $$y_{i}{}^{*}=\mu +\beta_{g}g_{i}+\epsilon_{i}$$

Where *β*_*g*_ is the marker regression coefficient (i.e., the allele substitution effect of the SNP) and *g*_*i*_ is the genotype (0, 1 or 2) of the sire i. For each SNP, *β*_*g*_ and its respective standard error (*SE*_*g*_) were estimated using ordinary least squares. The association between the SNP and the trait was assessed via a test statistic, calculated as:

(5) $$T^{2}=\frac{{\hat{\beta }}_{g}{}^{2}}{SE_{g}{}^{2}}$$

The test statistics are assumed to asymptotically follow a χ^2^ distribution with one degree of freedom under the null hypothesis. To assess the validity of this assumption, the deviation of the distribution of the test statistics from the expected theoretical quantiles was examined via 1) a quantile-quantile (Q-Q) plot, and 2) calculation of the inflation/deflation factor:

(6) $$\lambda =\frac{\mathrm{median}\left(T^{2}\right)}{0.456}$$

If λ < 1.1, the inflation was considered acceptable, and the Genomic Control (GC) correction was applied to adjust for that inflation [47]. Then, *P*-values were derived from the χ^2^ cumulative distribution function for the corrected test statistics. Finally, markers within the smallest 0.1% *P*-value percentile (i.e., most significant) were considered for re-analysis with the full model:

(7) $$y_{i}=\mu +\sum_{j=1}^{n}\beta_{j}X_{\mathrm{ij}}+\beta_{g}g_{i}+\epsilon_{i}$$

The EBVs were again weighted by their respective accuracies. The conservative Bonferroni adjustment for multiple testing (α=0.05/N, where N is the number of tests, i.e., number of SNPs) was used to reject the null hypothesis (*β*_*g*_ = 0, i.e. there is no association between the SNP and the EBVs), which resulted in an adjusted significance of α = 1.15 × 10^-7^.

### Exploratory view of significant SNPs

For any peak crossing the Bonferroni significance threshold, the estimated regression parameters were reported. For the most significant SNP, a 95% confidence interval (CI) for the estimated allele substitution effect size (${\hat{\beta }}_{g}$) was calculated, and the percentage of the EBV variance explained was calculated as:

(8) $$\%{\hat{\pi }}_{\sigma^{2}}=\frac{2\mathrm{pq}{\hat{\beta }}_{g}{}^{2}}{S^{2}}*100$$

Where *p* and *q* are the allele frequencies and *S*^*2*^ is the sample EBVs variance. The upper and lower limits of the estimated 95% CI for ${\hat{\beta }}_{g}$ were used to derive a 95% CI for % ${\hat{\pi }}_{\sigma^{2}}$.

The genomic region containing the most significant SNP of a peak was explored by inspecting a 1 Mb window around the location of this SNP using the *BioMart tool* and the *Ensembl genes 69* database [48] to interrogate 500 kb to each side of the marker using the UMD v3.1 assembly. The *cattle QTLdb* database [49] was also examined to find out if the significant SNP mapped against any previously described bovine QTL. Additionally, the alignments of the UMD v3.1 assembly sequence of the 1 Mb window against the human (*Homo sapiens*, GRCh37 assembly), pig (*Sus scrofa*, Sscrofa10.2 assembly) and mouse (*Mus musculus*, GRCm38 assembly) genome builds were inspected in the Ensembl *Comparative genomics alignments* and *Comparative genomics synteny* tools in order to determine if any homologous genes were present in the putative region.

## Abbreviations

BW: Birth weight; EBV: Estimated breeding value; SNP: Single nucleotide polymorphism; BTA: *Bos primigenius taurus*; GWAS: Genome-wide association study; IBS: Identity by state; MAF: Minor allele frequency; HWE: Hardy-weinberg equilibrium; PCoA: Principal coordinates analysis; Q-Q plot: quantile-quantile plot; GC: Genomic control; CI: Confidence interval; HSA: *Homo sapiens*; SSC: *Sus scrofa*; MMU: *Mus musculus*; QTL: Quantitative trait locus; CHILLP8: Rump fat thickness measured at the P8 position; QTN: Quantitative trait nucleotide; LD: Linkage disequilibrium

## Competing interests

The authors declare that they have no competing interests.

## Authors’ contributions

JFG conceived and led the coordination of the study. JS, JM, JBC, CPVT, FSS, MVGBS, LRPN and TSS contributed to the study design and coordination. ASC directed the genotyping work and contributed to the data analysis. RC and HHRN provided EBVs and assisted the data analysis. YTU led the data analysis and the manuscript preparation. MCM, LBZ and AMPO contributed in the data preparation and analysis. JBC, FSS, LRPN, TSS, CPVT, JFG, RC, HHRN, ASC and YTU interpreted the results and contributed to edit the manuscript. All authors read and approved the final manuscript.
